# Novel self-expandable metal stent with dumbbell-shape and spiral cover to prevent stent-related cholecystitis

**DOI:** 10.1055/a-2559-9546

**Published:** 2025-03-27

**Authors:** Haruo Miwa, Yugo Ishino, Shotaro Tsunoda, Ritsuko Oishi, Kazuki Endo, Yuichi Suzuki, Shin Maeda

**Affiliations:** 1 26437 Gastroenterological Center, Yokohama City University Medical Center, Yokohama, Japan; 2 Department of Gastroenterology, Yokohama City University Graduate School of Medicine, Yokohama, Japan

Covered self-expandable metal stents (cSEMS) are commonly used to treat malignant distal biliary obstruction (MDBO) 1 ; however, they can lead to cholecystitis 2 3 . A novel stent – the HILZO dumbbell-shaped stent with spiral cover (ABIS Inc., Hyogo, Japan) was designed to prevent stent-related cholecystitis 4 . The dumbbell-shaped proximal end creates space around the orifice of the cystic duct (OCD), while the spiral outer cover facilitates bile flow ( Fig. 1 , Fig. 2 ). Herein, we report two cases of MDBO in which the novel cSEMS was placed beyond the OCD ( Video 1 ).

**Fig. 1:**
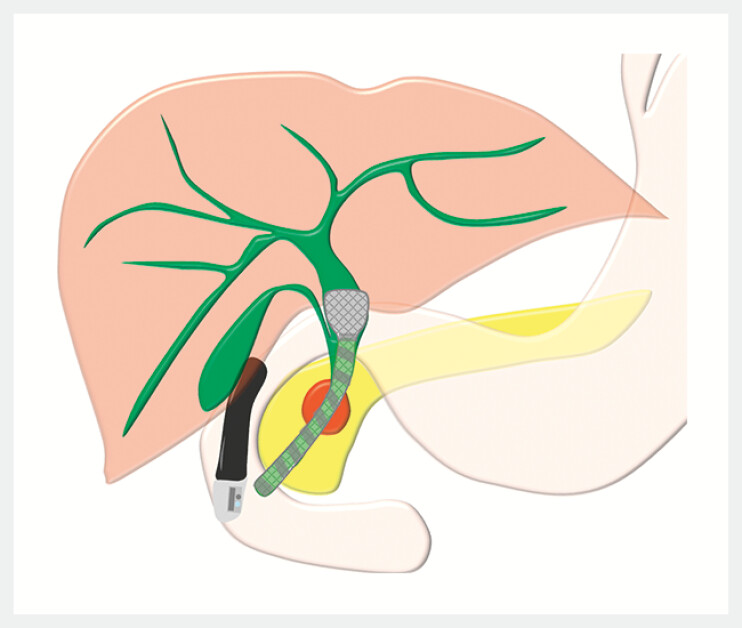
A novel HILZO dumbbell-shaped stent with spiral cover (ABIS Inc., Hyogo, Japan) is designed to prevent stent-related cholecystitis.

**Fig. 2:**
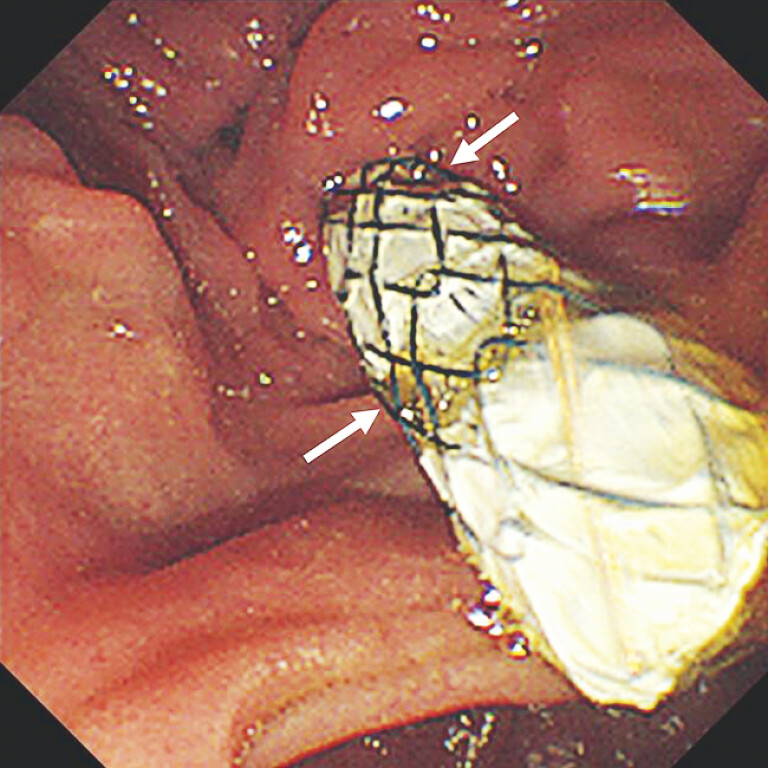
The spiral outer cover allows bile to flow out through the gaps (arrows).

The novel HILZO dumbbell-shaped stent with spiral cover (ABIS Inc., Hyogo, Japan) can prevent stent-related cholecystitis in patients with malignant distal biliary obstruction.Video 1

Case 1: A 62-year-old woman with MDBO secondary to pancreatic cancer was referred to our hospital. Endoscopic retrograde cholangiopancreatography was performed due to recurrent biliary obstruction after initial cSEMS placement. Cholangiography revealed the OCD above the biliary stricture. The novel cSEMS (8 mm, 7 cm) was deployed to cover the OCD with the covered part of the stent. Although contrast medium pooled in the cystic duct during the procedure, it flowed out and was replaced by air immediately after cSEMS placement ( Fig. 3 ). The patient was discharged without complications.

**Fig. 3:**
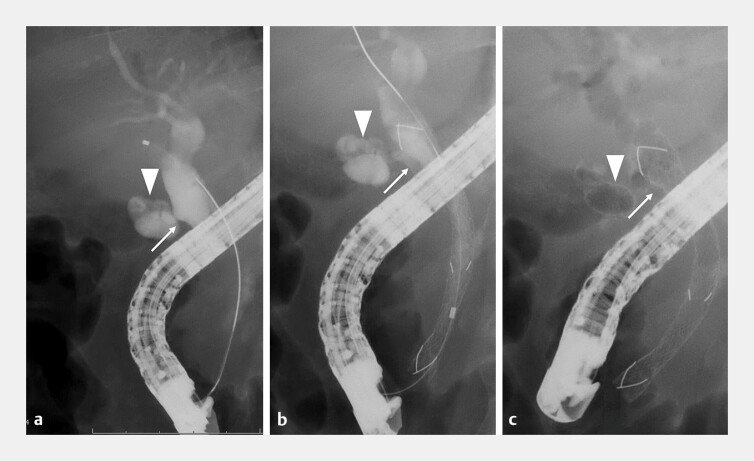
Case 1. **a** Cholangiography revealed the orifice of the cystic duct (arrows) above the stricture. **b** The novel HILZO stent (ABIS Inc., Hyogo, Japan) was deployed from the common hepatic duct to the duodenum. **c** Contrast agent that had pooled in the cystic duct flowed out after stent placement.

Case 2: An 80-year-old woman with MDBO due to pancreatic cancer was admitted to our hospital. Cholangiography revealed the OCD at the proximal end of the biliary stricture. Initially, transpapillary gallbladder drainage was attempted to prevent stent-related cholecystitis; however, a guidewire could not be advanced into the gallbladder due to the highly angulated cystic duct. Therefore, the novel cSEMS (8 mm, 7 cm) was placed beyond the OCD. Stent deployment was successfully performed from the common hepatic duct to the duodenum ( Fig. 4 ). The patient was discharged without any complications, and follow-up computed tomography showed a collapsed gallbladder compared with before cSEMS placement ( Fig. 5 ).

**Fig. 4:**
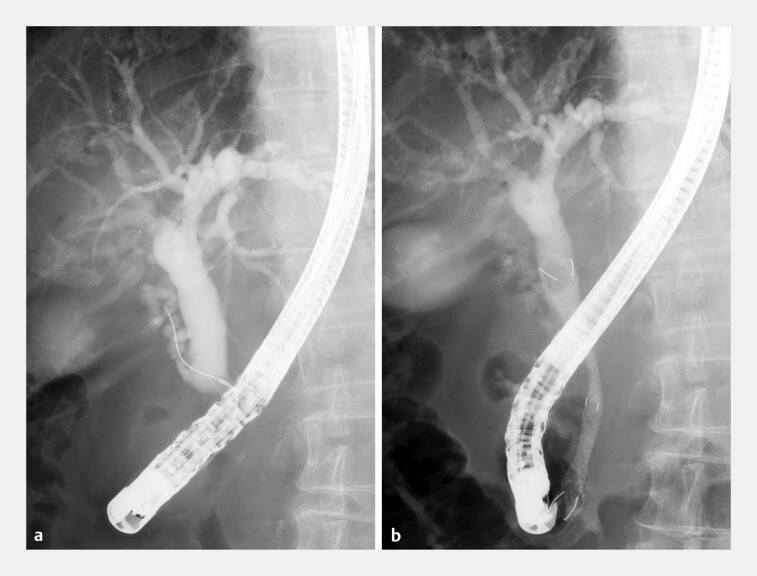
Case 2. **a** Cholangiography revealed the orifice of the cystic duct at the proximal end of the biliary stricture. **b** The novel HILZO stent (ABIS Inc., Hyogo, Japan) was successfully placed.

**Fig. 5:**
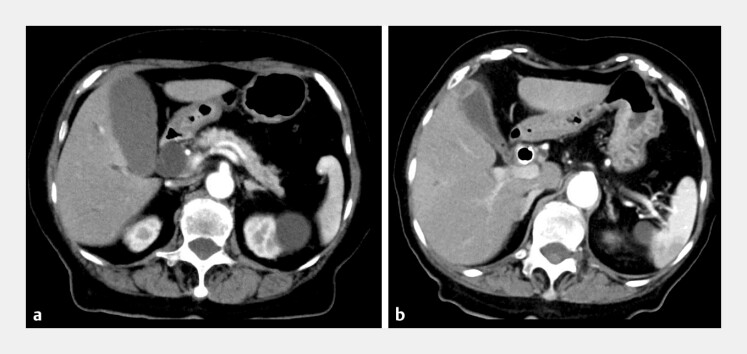
Case 2: Computed tomography showed the gallbladder collapse compared with before placement of the novel HILZO stent (ABIS Inc., Hyogo, Japan). **a** Before stent placement. **b** After stent placement.

To the best of our knowledge, this is the first report of a novel HILZO stent used to prevent stent-related cholecystitis in patients with MDBO.

Endoscopy_UCTN_Code_TTT_1AR_2AZ

## Notes

Endoscopy E-Videos https://eref.thieme.de/e-videos
*E-Videos* is an open access online section of the journal *Endoscopy* , reporting on interesting cases and new techniques in gastroenterological endoscopy. All papers include a high-quality video and are published with a Creative Commons CC-BY license. Endoscopy E-Videos qualify for HINARI discounts and waivers and eligibility is automatically checked during the submission process. We grant 100% waivers to articles whose corresponding authors are based in Group A countries and 50% waivers to those who are based in Group B countries as classified by Research4Life (see: https://www.research4life.org/access/eligibility/ ). This section has its own submission website at https://mc.manuscriptcentral.com/e-videos .
